# Post-Ganglionic Sympathetic Neurons can Directly Sense Raised Extracellular Na^+^ via SCN7a/Na_x_

**DOI:** 10.3389/fphys.2022.931094

**Published:** 2022-06-17

**Authors:** Harvey Davis, David J Paterson, Neil Herring

**Affiliations:** ^1^ Burdon Sanderson Cardiac Science Centre, Department of Physiology, Anatomy and Genetics, University of Oxford, Oxford, United Kingdom; ^2^ Wellcome Trust OXION Initiative in Ion Channels and Disease, Department of Physiology, Anatomy and Genetics, University of Oxford, Oxford, United Kingdom; ^3^ Oxford Heart Centre, Oxford University Hospitals NHS Foundation Trust, John Radcliffe Hospital, Oxford, United Kingdom

**Keywords:** stellate ganglia, sympathetic, dysautonomia, hypertension, sodium, nax

## Abstract

The relationship between dietary NaCl intake and high blood pressure is well-established, and occurs primarily through activation of the sympathetic nervous system. Na_x_, a Na^+^-sensitive Na^+^ channel, plays a pivotal role in driving sympathetic excitability, which is thought to originate from central regions controlling neural outflow. We investigated whether post-ganglionic sympathetic neurons from different ganglia innervating cardiac and vasculature tissue can also directly sense extracellular Na^+^. Using whole-cell patch clamp recordings we demonstrate that sympathetic neurons from three sympathetic ganglia (superior cervical, stellate and superior mesenteric/coeliac) respond to elevated extracellular NaCl concentration. In sympathetic stellate ganglia neurons, we established that the effect of NaCl was dose-dependent and independent of osmolarity, Cl^−^ and membrane Ca^2+^ flux, and critically dependent on extracellular Na^+^ concentration. We show that Na_x_ is expressed in sympathetic stellate ganglia neurons at a transcript and protein level using single-cell RNA-sequencing and immunohistochemistry respectively. Additionally, the response to NaCl was prevented by siRNA-mediated knockdown of Na_x_, but not by inhibition of other membrane Na^+^ pathways. Together, these results demonstrate that post-ganglionic sympathetic neurons are direct sensors of extracellular Na^+^ *via* Na_x,_ which could contribute to sympathetic driven hypertension.

## Introduction

Despite the relationship between dietary NaCl intake and hypertension being well established (Luft et al., 1979; Rose et al., 1988; Elliott et al., 1996; Neal et al., 2021), the mechanisms linking these have long been debated. Elevated NaCl intake or serum Na^+^ concentration following injection of a saline solution causes increased arterial blood pressure (Weiss et al., 1996; Fedorova et al., 2000; Ribeiro et al., 2015; Nomura et al., 2019) and sympathetic nervous system activity (SNA) in both animal models (Weiss et al., 1996; Ribeiro et al., 2015; Nomura et al., 2019) and humans (Farquhar et al., 2006). Prior work has established a causal link between the increased SNA and raised blood pressure following elevated serum NaCl concentration (Nomura et al., 2019).

The Na^+^-sensitive ion channel, Na_x_ (encoded by the gene *SCN7A*), acts as a direct molecular sensor of elevated extracellular Na^+^ (Hiyama et al., 2002). Interestingly, Na_x_ channels lack voltage-dependence and instead activate following an increase in extracellular Na^+^ concentration within the physiological range, which results in subsequent membrane depolarization (Hiyama et al., 2002). Emerging evidence reports a role of Na_x_ in central neurons and glia, in particular, the organum vasculosum of the lamina terminalis (OVLT) (Nomura et al., 2019) and subfornical organ (Hiyama et al., 2013). Currently, global Na_x_ knockout studies have highlighted a role in regulating salt intake, arterial blood pressure and recovery from peripheral nerve injury. Following Na_x_ knockout mice showed no preference for lower Na^+^ solutions following dehydration, unlike their wildtype counterparts (Watanabe et al., 2000). In relation to arterial blood pressure, global Na_x_ (*SCN7A*) knockout prevents the increase in SNA and corresponding hypertensive response to elevated dietary and plasma Na^+^ (Nomura et al., 2019).

The role of Na_x_ in the periphery is less well known and to our knowledge the contribution of Na_x_ to the function of sympathetic neuron function has not yet been investigated. In a study of sciatic nerve regeneration, Na_x_ knockout delayed recovery of sciatic nerve function following transection and this was suggested to be via the loss of Na_x_ mediated release of lactate from non-myelinating schwann cells (Unezaki et al., 2014). However, given that Na_x_ mediates the majority of the arterial blood pressure response to increased Na^+^ concentration through an increase in sympathetic nerve activity, we felt it was conceivable that there is also a role for peripheral Na_x_, in addition to central Na_x_ in mediating the increase in SNA following elevated extracellular Na^+^.

Using a combination of patch-clamp electrophysiology, live-cell imaging, single-cell RNA-sequencing, immunohistochemistry and gene expression knockdown, we tested the hypothesis that post-ganglionic sympathetic stellate ganglia neurons themselves behave as Na^+^ sensors, where hypernatremia induces a direct increase in their firing rate via Na_x_.

## Meterials and Methods

### Animals

Animal use complied with the University of Oxford Local Ethical Guidelines, the Guide for the Care and Use of Laboratory Animals (United States National Institutes of Health, Publication No. 85–23, revised 2011) and the Animals (Scientific Procedures) Act 1986 (United Kingdom). Animal experiments were approved and regulated under British Home Office Project Licenses (PPL 30/3131 and P707EB251). Rats were used as the chosen model in this study based upon the breadth of cardiovascular research on this model organism (as reviewed in Herring et al., 2019), and our preexisting single-cell RNA-sequencing dataset from the rat stellate ganglia (Davis et al., 2020). Rats were purchased from Envigo, United Kingdom Animals were housed in the local animal facility in open top conventional cages at 20–24°C with 55% Relative Humidity on a 12-h day-night cycle. Rats were anesthetized via isoflurane and euthanized by an intraperitoneal overdose of pentobarbitone and confirmed by exsanguination according to Schedule one of the Animals (Scientific Procedures) Act 1986 (United Kingdom).

### Cell Dissociation and Culture

To produce neuronal cell cultures, a specified sympathetic ganglia was dissected from recently euthanized 4-5-week-old male Wistar rats. 4–5-week-old rats were used for this study, as a compromise between the cell yield and cell viability for primary sympathetic neuron culture which is improved in younger rats. Male rats were used to avoid potential confounding effects from previously established sex differences between male and female stellate ganglia (Bayles et al., 2019), with males chosen due to the higher rate of hypertension incidence in men compared to women prior to menopause (Reckelhoff, 2001). Following euthanasia ganglia were immediately placed into ice-cold L-15 media. Contaminating tissue and fibrous sheaths surrounding the ganglia were then visually identified and removed. Tissue was cut into roughly 1 mm long sections, and then maintained at 37°C in an agitator water bath for 25 min in L-15 media with 1 mg/ml collagenase IV (Worthington, United States). Cells were then incubated for a further 30 min in Ca^2+^ and Mg^2+^ free hanks buffered salt solution with 2 mg/ml Trypsin (Worthington, United States) with 0.1 mg/ml DNAase I (Fischer, 1982) to reduce cytotoxic cell aggregation (Reichard and Asosingh, 2019). Once enzymatic digestion was complete, tissue was immediately transferred to a fetal bovine serum (FBS) based blocking solution containing 5% FBS, 100 Units/mL) penicillin-streptomycin and neurobasal plus medium. The cell suspension was then plated onto poly-d-lysine precoated Fluorodish 35 mm dishes (WPI, United States) which had been pre-incubated for 2 hours with 1 μg/ml laminin, a concentration chosen to limit neuronal outgrowth whilst still promoting cell survival (Buettner and Pittman, 1991). Cell containing dishes were then incubated at 37°C with 95% oxygen and 5% CO_2_ for a period of one to 5 days. For most experiments, cells were plated with Neurobasal plus media (Thermofisher, United States) with B27 plus supplement (Thermofisher, United States) and glutamax (Thermofisher, United States), supplemented with an additional 50 Units/ml penicillin/streptomycin (Sigma-Aldrich, United States) and 100 ng/ml nerve growth factor (Merck-Millipore, United States).

### Patch-Clamp Electrophysiology

Cells were allowed to equilibrate for at least 30 s, before recording was commenced. The extracellular solution for whole-cell patch-clamp recordings contained: 5.2 mM KCl, 140 mM NaCl, 1 mM MgCl_2_, 1.8 mM CaCl_2_, 10 mM HEPES, 10 mM Glucose. The internal solution contained: 130 mM K^+^ -Gluconate, 10 mM KCl, 10 mM HEPES, 10 mM Na^+^ -Phosphocreatine, 4 mM MgATP, 0.3 mM Na_2_GTP. Internal pH was adjusted to 7.3 with KOH. An agar-KCl salt bridge was used as a grounding electrode. Data were acquired using WinWCP (Versions 5.4.0–5.5.4) and recorded via a Multiclamp 700B amplifier (Molecular Devices, United States) and an axon digidata 1550A (Molecular devices, United States) digitizer. All recordings were performed at room temperature. Pipettes with a tip resistance of 2–4 MΩ were used. A calculated liquid junction potential of 16.2 mV via the implementation of JPCalC in Clampex Version 11.1.0.23 (Molecular Devices, United States) was not subtracted, due to the focus upon relative changes rather than absolute membrane potential. Following correction these data would sit within the previously reported resting membrane potential range (Davis et al., 2020). Current-clamp recordings with a R_s_ greater than >20 MΩ were discarded. Current-clamp recordings with a R_s_ change greater than 30% between the start and the end of the recording period were discarded. Cells with a leak current > 90 pA were discarded. Where applicable, drugs were added for a minimum of 3 min prior to the addition of 170 mM NaCl and paired comparisons were made between drug-treated cells and drug-treated cells in 170 mM NaCl. Drugs, hyperosmotic or hypernatraemic solutions were applied or washed out for 3 min *via* bath perfusion before recording firing rate changes. Firing rate was calculated as the maximum firing rate induced by a series of current injections between 0–200 pA, a range that we previously found to elicit a maximal firing rate response in cultured stellate ganglia neurons which do not typically exhibit firing at rest (Davis et al., 2020).

### Intracellular Na^+^ Imaging

Na^+^-binding benzofuran isophthalate acetoxymethyl ester (SBFI-AM) was utilized to image changes in intracellular Na^+^ concentration as previously described for the study of Na_x_ in other cell types (Hiyama et al., 2002). A stock solution of 1 mM SBFI-AM was prepared in DMSO with 20% pluronic acid F127 and then loaded at 10 μM for a period of 1.5–2 h at room temperature. SBFI was alternatively stimulated at wavelengths 355 nM and 380 nM, and emission was detected at 510 nM. Calibrations were performed in solutions containing 5 μM gramicidin D, 10 μM monensin, and 1 mM ouabain with solutions containing, 0 mM, 10 mM, 20 mM, 30 mM or 40 mM NaCl. Each NaCl concentration was prepared by mixing two solutions containing either 135 mM NaCl or 135 mM KCl, alongside 10 mM HEPES, 2 mM EGTA. Solutions were buffered with either NaOH or KOH.

### Intracellular Ca^2+^ Imaging

Cells were incubated for 30 min at 37°C in 2 μM Fura-2 AM dissolved in extracellular solution. In brief, following incubation with Fura-2, cells were washed three times in extracellular solution. The 100 µL recording chamber was maintained at 36 ± 0.5°C *via* an inline heater under constant gravity-fed perfusion at a rate of 5–6 ml/min. Images were acquired with a QICLICK digital CCD camera (Photometrics, United States). Fura-2 was alternatively stimulated at wavelengths 355 nM and 380 nM, and emission was detected at 510 nM. Recordings were discarded where significant drift was observed in the baseline ratio, or the baseline Fura-2 ratio differed by > 1.5x the interquartile range for all baseline values. Fluorescence values were determined by averaging whole cell fluorescence with background fluorescence subtracted. Baseline was taken as the average ratio over a 30 s period proceeding to drug perfusion or solution changes. Average responses were measured as an average of at least ten points.

### Single-Cell RNA-Sequencing

Single-cell suspensions were prepared via enzymatic and mechanical dissociation as described above. The single cell suspension was then resuspended in phosphate-buffered saline with fetal bovine serum and then sequenced by 10x Genomics based single cell RNA-sequencing via the Wellcome Trust Centre for Human Genetics, Oxford. Data were aligned using the 10x genomics pipeline (Zheng et al., 2017), which utilizes a star-based alignment methodology (Dobin et al., 2013) as described previously (Davis et al., 2020). The single-cell sequencing dataset used for this study is available at genome expression omnibus (GSE144027).

### Immunohistochemistry

Cells were cultured as described for 48 h, before fixation for 10 min in 4% paraformaldehyde, followed by three 30 s washes in phosphate buffered saline (PBS). Cells were then blocked and permeabilized in 0.1% triton-x, with donkey serum and 3% BSA for 30 min. Cells were subsequently washed three further times in PBS. Cells were co-labelled with rabbit anti-SCN7A (TA314512, Origene, United States) and sheep anti-TH (PA14679, Life technologies, United States) primary antibodies for a period of an hour at 37°C. Cells were washed three times in PBS before anti-rabbit Alexa fluor 594 and anti-sheep Alexa Fluor 488 were used as secondary antibodies. Cells were again washed three times in PBS before cell nuclei counterstaining with Hoescht 33342 stain for 1 minute and washed three further times in PBS before images were acquired at 40x magnification on an inverted Nikon microscope.

### siRNA-Mediated Knockdown

siRNA knockdown of either *GAPDH* (s236443)*,* a scrambled sequence of RNA (4390843) or *SCN7A* (s133825) was achieved via pre-designed Silencer assays (ThermoFisher, United States). Cells were transfected through lipofection via RNAiMAX (ThermoFisher, United States) and incubated with the lipofectamine-siRNA mixture for a period of 2–3 days before recording. siRNA was prepared in Optimem I (Thermofisher, United States) serum free media, alongside Lipofectamine RNAiMax (Thermofisher, United States) to achieve a final concentration of 10 nM siRNA per well in 1 ml of culture medium.

### Analysis and Statistics

Data were normality tested via an Anderson-darling test, D’Agostino-Pearson test, Shapiro-Wilk test, and Kolmogorov-Smirnov test as well as visual inspection of QQ plots. If ≥ 3 tests and the QQ plot suggested normality, then parametric statistics were used, otherwise appropriate non-parametric analyses were adopted. Statistical analyses were performed in GraphPad Prism. Unless otherwise specified, n refers to cell number, with ≥2 cultures used, with each culture using a minimum of two animals. Membrane potentials are shown in text as mean ± standard error of the mean, maximum firing rate is shown as the median value. A *p*-value <0.05 was chosen to indicate significance.

## Results

### Sympathetic Neurons can Respond to Elevated Extracellular NaCl

**FIGURE 1:**
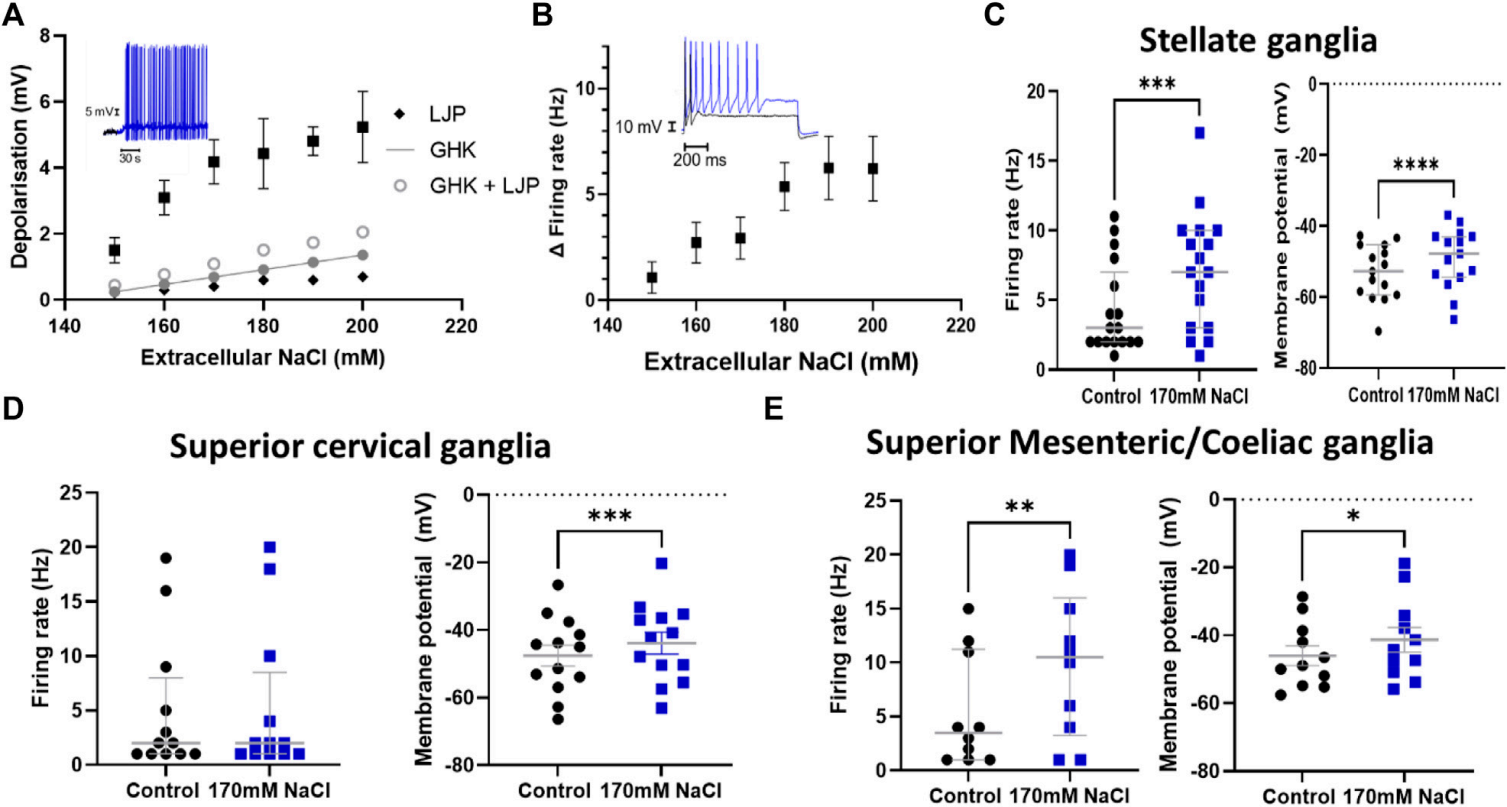
Sympathetic stellate ganglia neurons are excited by acute elevated NaCl. **(A)** The effect of [NaCl]_o_ on stellate ganglia neuron membrane potential (Black) is dose dependent between 150–200 mM and is greater than that predicted by the calculated change in liquid junction potential (Black diamond), Goldman-Hodgkin-Katz equation (Closed grey circles), or the combined Goldman-Hodgkin-Katz equation and calculated change in liquid junctional potential (Open grey circles). Inset data trace shows membrane depolarization and resulting spontaneous firing following 170 mM [NaCl]_o_ (Blue) following an initial period in 140 mM NaCl (Black) (*n* = 12–22; Welch’s ANOVA test, *p* < 0.0001). **(B)** The increase in firing rate following an increase in extracellular Na^+^ concentration is shown as Mean ± SEM (n = 8–18; Welch’s ANOVA, *p* = 0.01). The inset trace shows the evoked response of a stellate ganglia neuron to 150 pA current injection before and after treatment with 170 mM NaCl. The black trace represents a recording at 140 mM NaCl, the blue trace is following 170 mM NaCl. **(C)** The addition of 170 mM NaCl significantly depolarized cultured stellate ganglia neurons and caused an increase in maximum induced firing. 170 mM NaCl increased neuron firing (3 Hz–7 Hz; n = 17; Wilcoxon test; *p* = 0.0009) (Left) and induced stellate ganglia neuron membrane depolarization (4.07 ± 0.75 mV; n = 15; Paired *t*-test; *p* < 0.0001) (Right) and. **(D)** An increase in extracellular NaCl to 170 mM also significantly depolarized cultured superior cervical ganglion neurons (3.68 ± 0.78 mV; n = 13; Paired *t*-test; *p* = 0.0005) (Right), but did not significantly increase their stimulated maximum firing rate (19 Hz–20 Hz; n = 12; Wilcoxon test; *p* = 0.48) (Left). **(E)** Increasing extracellular NaCl to 170 mM caused significantly increased neuronal firing rate (3.5 Hz–10 Hz; n = 10; Wilcoxon test; *p* = 0.008) (Left) and a significant depolarization of superior mesenteric/coeliac ganglia resting membrane potential (4.72 ± 1.53 mV; n = 11; Paired *t*-test; *p* = 0.01) (Right).

We first investigated whether post-ganglionic sympathetic neurons could respond to changes in extracellular NaCl concentration ([NaCl]_o_). Using whole-cell patch-clamping we observed a significant dose-dependent membrane depolarization following a change in [NaCl]_o_ from 140 mM to 150–200 mM in stellate ganglia neurons (Figure 1A). These concentrations were chosen based upon prior measurements of elevated serum Na^+^ concentration in rodent models with a high salt diet or DOCA-salt induced hypertension (Nomura et al., 2019). The experimental relationship between extracellular NaCl and membrane depolarization was observed to be greater than the linear relationship predicted by the calculated change in liquid junction potential (Black diamond), Goldman-Hodgkin-Katz equation, or for the combined Goldman-Hodgkin-Katz equation and calculated change in liquid junctional potential. The membrane depolarization observed in Figure 1A, was also associated with a significant increase in maximum neuronal firing rate (Figure 1B). Having established that stellate ganglia neurons can respond to the full range of concentrations used (150–200 mM), we used 170 mM, the lowest concentration where the membrane potential and firing rate response plateaus for further experiments. This concentration parallels the concentration used in previous studies of Na_x_ but also provides a robust effect for subsequent pharmacological dissection of the underlying mechanism. In stellate ganglia neurons an increase in [NaCl]_o_ to 170 mM had a significant effect upon the membrane potential and induced firing rate (Figure 1C). We observed similar results in response to 170 mM [NaCl]_o_ in sympathetic neurons from the superior cervical ganglia (Figure 1D) and the superior mesenteric/coeliac ganglia (Figure 1E) in terms of membrane potential depolarization, although superior cervical ganglia neurons did not exhibit an increase in firing rate. For subsequent mechanistic study we focused upon stellate ganglia neurons, which exhibited both a membrane depolarization and firing rate effect in response to elevated [NaCl]_o._

### The Response to Raised Extracellular Na^+^ Is Independent of Changes in Osmolarity

**FIGURE 2:**
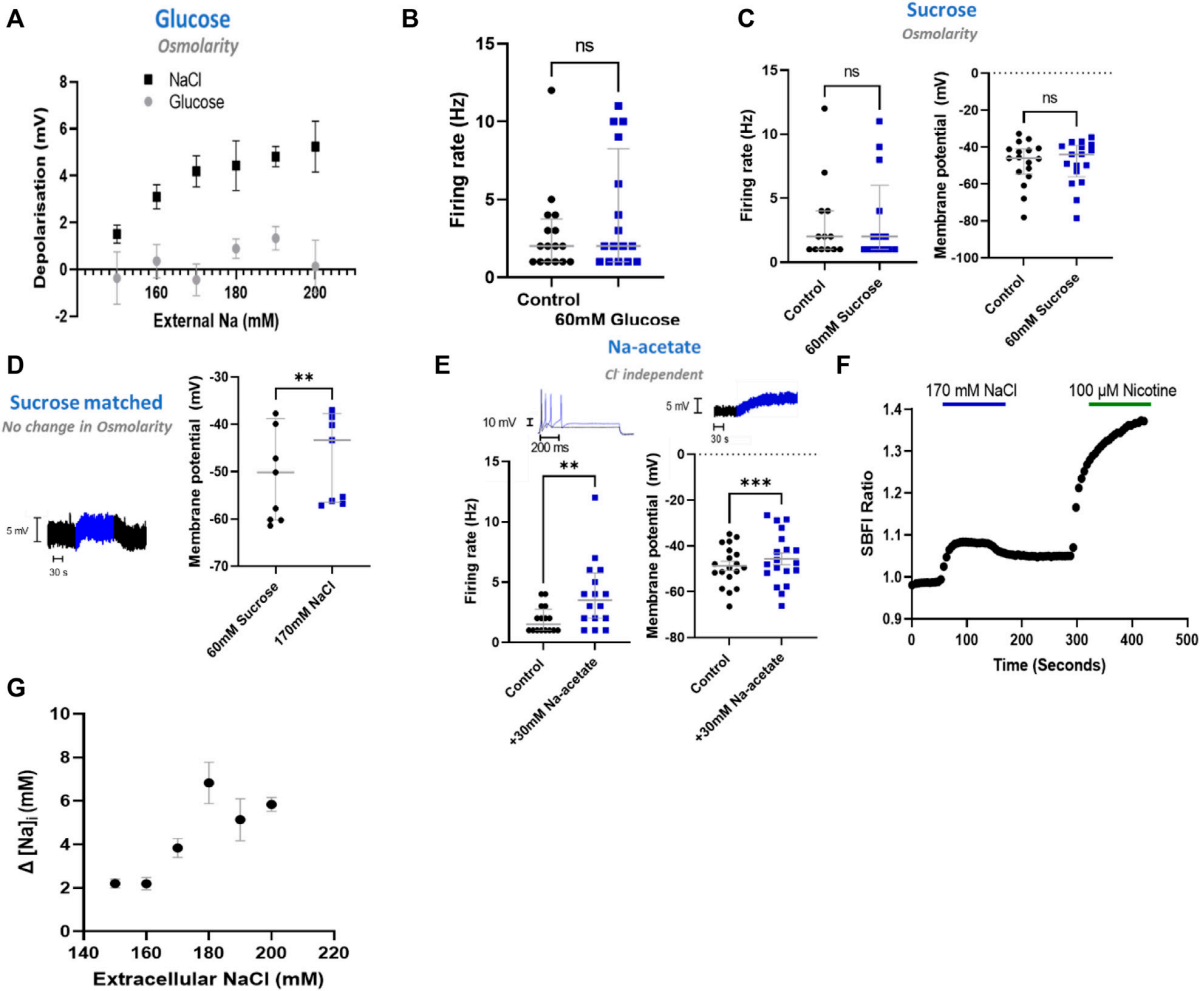
NaCl evokes an effect in stellate ganglia neurons independent of osmolarity. **(A)** Osmolarity matched glucose controls did not induce membrane depolarization like NaCl. Over the range 20 mM–120 mM glucose, osmolarity matched NaCl caused a significantly greater depolarization than tested concentrations of glucose (Two-way ANOVA; *p* < 0.0001) (NaCl, n = 15–22; Glucose, n = 8–15). **(B)** The addition of 60 mM glucose had no statistically detectable effect upon neuronal firing. 60 mM glucose did not significantly alter stellate ganglia neuron firing (n = 16; Wilcoxon test; *p* = 0.09). **(C)** 60 mM sucrose had no effect upon induced neuronal firing or membrane potential. Firing rate was unaltered following the addition of 60 mM sucrose (n = 13; Wilcoxon test; *p* = 0.50), as was the neuronal membrane potential (n = 17; Paired *t*-test; *p* = 0.95). **(D)** 170 mM NaCl induced a response from osmolarity matched solution high sucrose solution. Transitioning from 60 mM sucrose control solutions to 170 mM NaCl in the presence of 1 µM TTX still elicited a membrane depolarization despite balanced osmolarity (n = 9; Paired *t*-test; *p* = 0.005). **(E)** The response was not Cl^−^dependent as 30 mM Na-acetate increased stellate ganglia neuron firing rate (1.5 Hz–3.5 Hz; *n* = 14; Wilcoxon test; *p* = 0.02) and depolarized the membrane potential (3.08 ± 0.71 mV; n = 17; Paired *t*-test; *p* = 0.002). Firing rate is shown before (black) and after (blue) 30 mM Na-Acetate (Left trace). Membrane depolarization is shown following 170 mM NaCl. **(F)** A raw SBFI data trace, showing the response to 170 mM NaCl (Blue) and 100 µM nicotine (Green) for comparison. **(G)** Elevated [NaCl]_o_ caused a dose-depednent increase in intracellular Na^+^ concentration ([Na^+^]_i_) (Kruskal Wallis test, *p* < 0.0001), as measured with the Na^+^ selective dye SBFI. Dye ratios were converted to changes in NaCl, via the calibration curve in extended data Figure 1.

Having determined that NaCl has a direct effect upon sympathetic neuron electrophysiology in stellate ganglia, superior cervical ganglia and superior mesenteric/coeliac ganglia derived neurons, we then proceeded to investigate the mechanism using stellate ganglia neurons. First, we interrogated whether this effect was mediated through changes in osmolarity. To do so, we employed four approaches, first in Figure 2A we demonstrated that glucose had no dose-dependent effect upon membrane depolarization when osmolarity matched with changes in [NaCl]_o_ (+20–120 mM glucose). The addition of 60 mM glucose, which is osmotically equivalent to a 30 mM increase in [NaCl]_o_, had no detectable effect upon firing rate as shown in Figure 2B. Similarly, 60 mM sucrose, had no detectable effect upon the neuronal membrane potential or maximum firing rate as demonstrated in Figure 2C.

To confirm these data, we repeated the increase of [NaCl]_o_ to 170 mM but starting from osmolarity matched solutions (+60 mM sucrose) (Figure 2D). These experiments highlight that 170 mM [NaCl]_o_ still causes a statistically significant membrane depolarization despite constant osmolarity. For these recordings the response was not significantly different from 170 mM added in non-osmotically matched solutions (One-tailed unpaired *t*-test; *p* = 0.30).

### The Response of Sympathetic Stellate Ganglia Neurons to Elevated NaCl Is Na^+^ Dependent

To investigate whether the effect of stellate ganglia neurons to NaCl in Figures 1A–C was Na^+^ dependent and Cl^−^ independent, two approaches were taken. In the first, presented in Figure 2E, additional Na-acetate (30 mM) was added in lieu of NaCl. The addition of 30 mM Na-acetate also caused a depolarisation and increased firing rate. Further analysis, highlights that the change observed with Na-Acetate is not significantly smaller than that observed with NaCl (Membrane potential, Unpaired-test, *p* = 0.26; Firing rate, Mann Whitney test; *p* = 0.62). A Na^+^-dependent mechanism is similarly suggested by a second approach, in which we utilized SBFI, a ratiometric Na^+^ sensitive dye, to image changes in intracellular Na^+^ concentration following an increase in extracellular NaCl concentration. Raw SBFI data were converted to changes in intracellular Na^+^ concentration using the calibration curve shown in Extended Data 1. These data, shown in Figures 2F,G, show that increases in [NaCl]_o_ cause a rapid increase in intracellular Na^+^ concentration, in a dose dependent manner. A typical response to 170 mM NaCl is shown in Figure 2F, with averaged responses to 150 mM–200 mM shown in Figure 2G.

**EXTENDED DATA 1:**
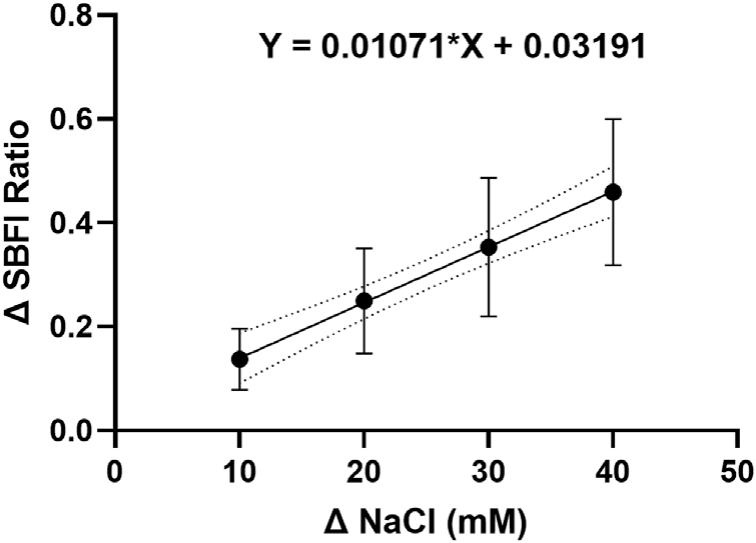
The SBFI calibration curve indicates a linear relationship between the intracellular sodium ion concentration and the change in dye fluorescence.

### The NaCl Response in Stellate Ganglia Neurons Is ENaC, Na+/K + ATPase and Na_v_ Independent

**FIGURE 3:**
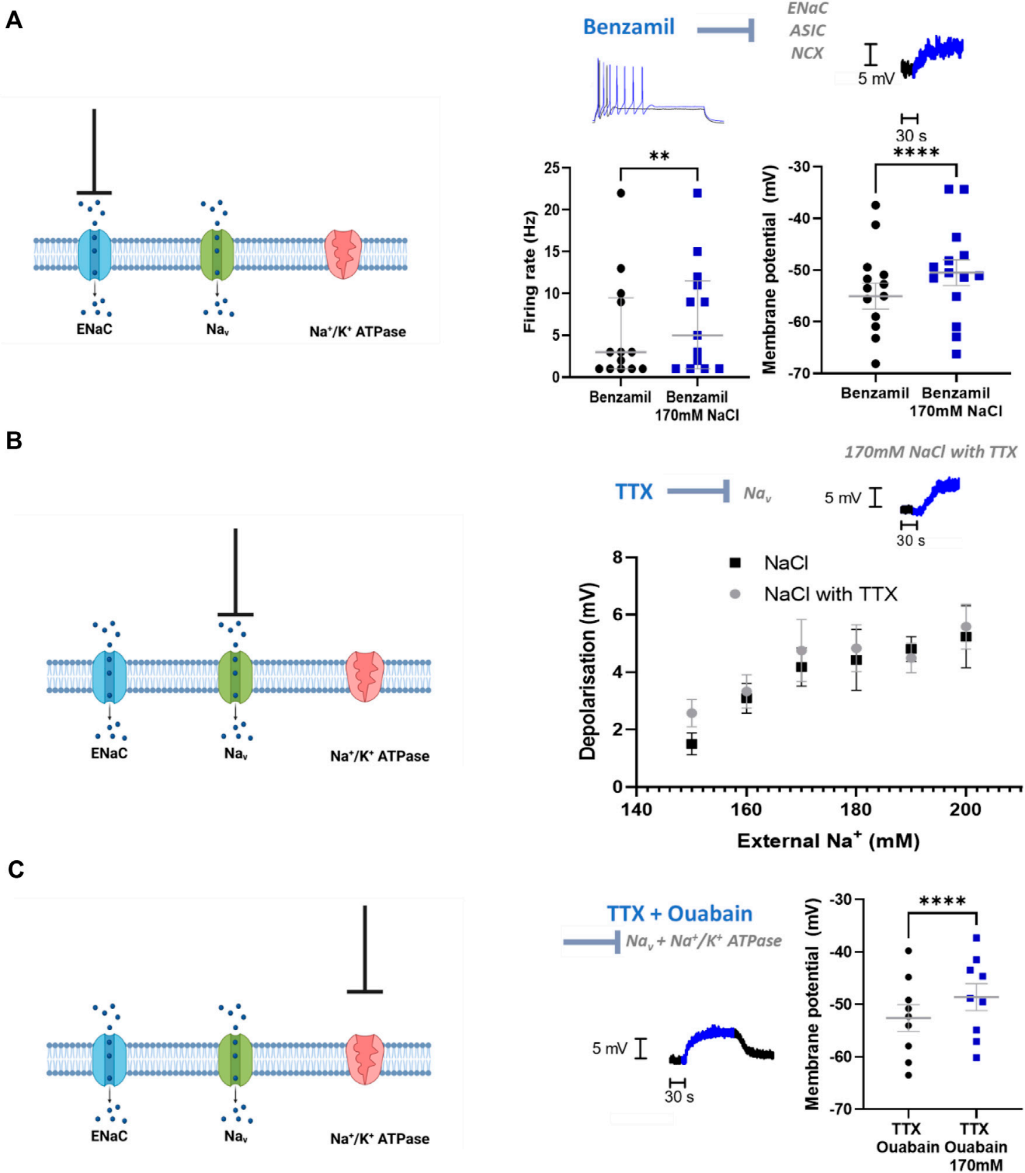
ENaCs, Na_v_ and Na^+^/K^+^ ATPase do not contribute to the NaCl response in stellate ganglia neuron. **(A)** 1 µM Benzamil does not prevent NaCl induced firing rate increases or membrane depolarization in stellate ganglia neurons. In the presence of 1 µM Benzamil 170 mM NaCl still increased maximum firing rate (3 Hz–5 Hz; n = 13; Wilcoxon test; *p* = 0.0078) and caused a depolarization (4.55 ± 0.55 mV; n = 14; Paired *t*-test; *p* < 0.0001). For the raw data trace, 1 µM Benzamil with physiological [NaCl]_o_ is shown in black, and 1 µM Benzamil with 170 mM [NaCl]_o_ is shown in blue. **(B)** TTX had no effect upon membrane depolarization induced by 150–200 mM NaCl in stellate ganglia neurons There was no significant difference between membrane depolarization induced by NaCl in the presence or absence of 1 µM TTX. For raw data trace, physiological extracellular Na + shown in black and 170 mM NaCl with TTX is shown in blue (Control, n = 15–22; 1 µM TTX, n = 7–13; Two-way ANOVA; *p* = 0.42). **(C)** TTX and ouabain did not prevent 170 mM NaCl induced depolarization. 170 mM NaCl caused a significant depolarization of sympathetic neurons in the presence of both 1 µM TTX and 100 µM Ouabain relative to 1 µM TTX and Ouabain alone (3.98 ± 0.48 mV; n = 9; Paired *t*-test; *p* < 0.0001). On the raw data trace, baseline and washout are shown in black, 170 mM NaCl is shown in blue.

We then investigated the mechanism by which sympathetic stellate ganglia neurons respond to elevated extracellular Na^+^ concentration. Epithelial Na + channels (ENaCs) represent a major mechanism of Na^+^ transport across the membrane and conduct Na^+^ under resting conditions. ENaCs therefore represent a feasible mechanism by which Na^+^ influx could occur. ENaC inhibitors, benzamil and amiloride (Benzamil; Figure 3A) (Amiloride; Depolarization, *n* = 14, Wilcoxon matched pairs test; *p* = 0.002; Neuronal firing rate, n = 14, Paired *t*-test, *p* < 0.0001), did not prevent membrane depolarization or the increased firing rate following 170 mM [NaCl]_o_. In addition, these compounds did not reduce the membrane depolarization or increased firing rate induced by 170 mM [NaCl]_o_ (Benzamil; Depolarisation, n = 14–17, One-tailed unpaired *t*-test, *p* = 0.34; Neuronal firing rate, n = 14–17, One-tailed Mann-Whitney test, *p* = 0.14) (Amiloride; Membrane depolarization; n = 14–17, One-tailed unpaired *t*-test, *p* = 0.22; Neuronal firing rate, n = 14–17, One-tailed Mann-Whitney test, *p* = 0.33).

At the tested concentration benzamil also inhibits acid sensitive ion channels (ASICs) (Leng et al., 2016) and the Na^+^/Ca^2+^-exchanger (NCX) (Wang et al., 2008), therefore these mechanisms are also excluded by these data.

Voltage-gated Na^+^ channels (VGSCs) are unlikely to be activated at physiological membrane potentials, but it is possible that a small proportion of channels are active in the persistent conductance state, which lacks inactivation. TTX-resistant VGSCs are not present in stellate ganglia neurons (Davis et al., 2020) and so in Figure 3B we used TTX to block all VGSCs present in stellate ganglia neurons. Whilst TTX prevents the recording of firing rate changes, this experiment allowed us to measure the effect on membrane potential in response to raised external NaCl. We observed no significant difference between increasing [NaCl]_o_ in the presence or absence of TTX (Figure 3B), suggesting that VGSCs are not involved in this response.

Na^+^/K^+^ ATPase is responsible for determining resting Na^+^ gradients through the mass transport of Na^+^. To assess the contribution of Na^+^/K^+^ ATPase to the observed Na^+^ influx with elevated [NaCl]_o_, we inhibited Na^+^/K^+^ ATPase *via* ouabain (Figure 3C). These experiments were performed in the presence of the VGSC inhibitor TTX (1 µM), as ouabain alone caused the cells to spontaneously fire, which made the measurement of the membrane potential challenging. Ouabain (100 µM) did not prevent 170 mM [NaCl]_o_ induced membrane depolarization beyond that caused by ouabain alone, and this effect size was not observed to be significantly reduced compared to control (One-tailed unpaired *t*-test; *p* = 0.42).

### The Mechanism Is Ca^2+^ Independent

**FIGURE 4:**
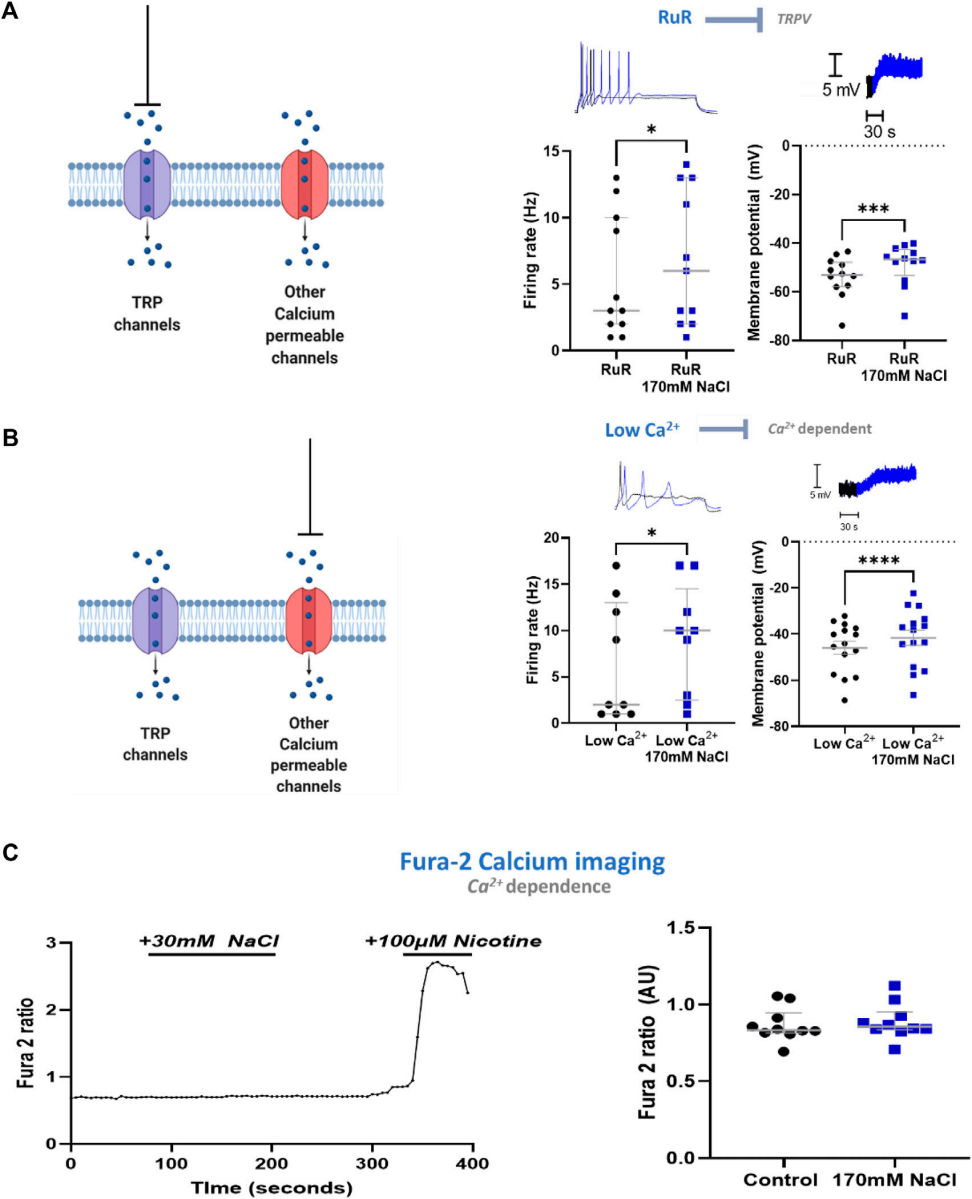
The excitatory response to NaCl is independent of Ca^2+^ in stellate ganglia neurons. **(A)** TRPV channel inhibition by ruthenium red does not prevent the effects of high NaCl. TRPV inhibitor ruthenium red (10 µM) did not prevent 170 mM induced increased maximum firing frequency (3 Hz–6 Hz, Wilcoxon matched-pairs test, *p* = 0.02) nor membrane depolarization (5.14 ± 1 mV, Paired *t*-test, *p* = 0.0003). **(B)** Reducing external Ca^2+^ from 1.8 mM to 0.2 mM did not prevent the effect of 170 mM NaCl. When recording in low Ca^2+^ Tyrode’s solution 170 mM NaCl still increased neuronal firing (2 Hz–10 Hz; n = 9; Wilcoxon test; *p* = 0.031) and evoked membrane depolarization in sympathetic stellate ganglia neurons (4.32 ± 0.72 mV; *n* = 15; Paired *t*-test; *p* < 0.0001). For the raw data trace, low extracellular Ca^2+^ concentration with physiological [NaCl]_o_ is shown in black, low extracellular Ca^2+^ concentration with 170 mM [NaCl]_o_ is shown in blue. **(C)** High [NaCl]_o_ does not evoke a Ca^2+^ response in stellate ganglion neurons. 170 mM [NaCl]_o_ evoked no significant intracellular Ca^2+^ response in sympathetic stellate ganglia neurons as assessed by Fura-2 live-cell Ca^2+^ imaging (*n* = 10; Paired *t*-test; *p* = 0.066). Control recordings were taken as the average between 20–60 s of recording, the response was taken as the peak value between 80–200 s of recording. Nicotine was added to demonstrate that the tested stellate ganglia neurons were responsive to external stimuli. All tested neurons had a nicotinic response comparable to that shown here.

Having established that the mechanism in stellate ganglia neurons is Cl^−^ independent, osmolarity independent, and independent of ubiquitous Na^+^ mechanisms, we explored whether the mechanism involved Ca^2+^ or a Ca^2+^ associated process. We first investigated transient receptor potential (TRP) channels, which are associated with physiological responses to altered osmolarity and have previously been linked to hypothalamic NaCl responses (Naeini et al., 2006). For example, TRPV2 and TRPV3 have been described in sympathetic ganglia. TRP channel inhibition by either 10 µM ruthenium red (RuR), a vanillin family TRPV channel inhibitor (Figure 4A) or 50 µM Gd^3+^, a non-selective TRP channel inhibitor failed to prevent a statistically significant membrane depolarization following an elevation of extracellular NaCl to 170 mM in stellate ganglia neurons (3.61 ± 0.84 mV, Paired *t*-test, *p* = 0.003). Further analysis highlighted that the extent of depolarization was not reduced by these inhibitors (RuR, One-tailed unpaired *t*-test, *p* = 0.21; Gd^3+^, One-tailed unpaired *t*-test; *p* = 0.30). RuR did not prevent increased neuronal firing following 170 mM NaCl in stellate ganglia neurons (Figure 4A), and the effect of 170 mM NaCl upon stellate ganglia neuron firing rate was not found to be smaller in the presence of RuR (One-tailed Mann-Whitney test; *p* = 0.13). The effect of firing rate was not tested with Gd^3+^, as Gd^3+^ alone prevented neuronal firing.

In stellate ganglia neurons we then excluded a contribution of other Ca^2+^-dependent mechanisms by reducing extracellular Ca^2+^ concentration to 0.1 mM. Under this condition, 170 mM NaCl still induced statistically significant membrane depolarization and increased neuronal firing rate (Figure 4B). These effects were found to not be statistically reduced compared to the effect of 170 mM NaCl on neurons exposed to physiological extracellular Ca^2+^ concentration (2 mM) (Membrane depolarization, One-tailed unpaired *t*-test, *p* = 0.45; Neuronal firing rate, One-tailed Mann-Whitney test, *p* = 0.17).

Finally, we used Fura-2 AM based Ca^2+^ imaging (Tsien, 1986) to confirm the absence of a Ca^2+^ influx following elevated NaCl. Using stellate ganglia neurons, this methodology confirmed that there was no detectable increase in intracellular Ca^2+^ concentration (Figure 4C), and it is unlikely that a large Ca^2+^ conductance is contributing to this mechanism. At the end of the recording period, the sympathetic neuron agonist nicotine was added to demonstrate that the neurons remained responsive.

### In Stellate Ganglia Neurons Na^+^ Sensitivity Is Conferred by Na_x_

**FIGURE 5:**
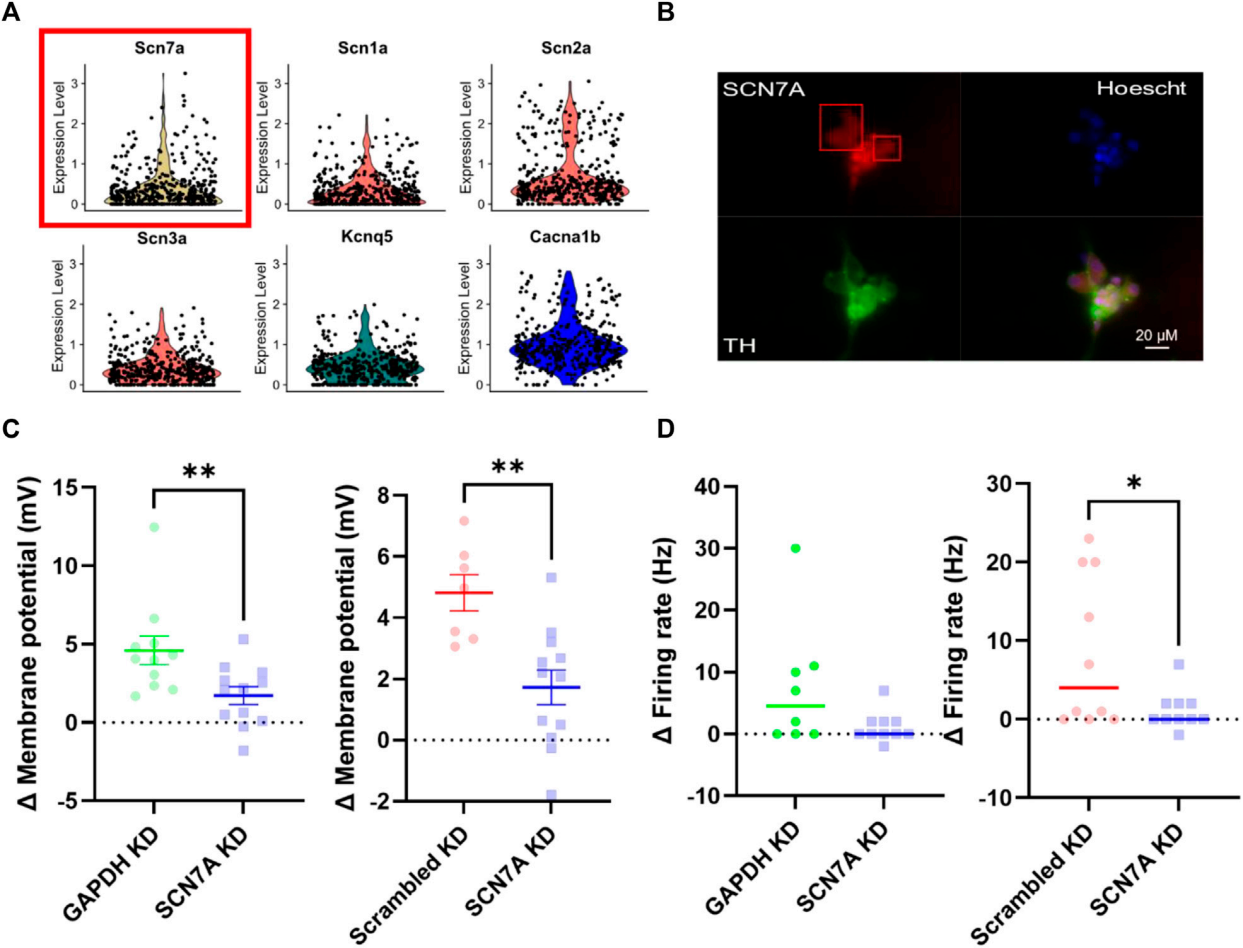
Na_x_ is responsible for sodium sensitivity in stellate ganglia neurons. **(A)** Single-cell RNA-sequencing highlighted expression of the Na_x_ encoding gene SCN7A (Yellow, with red box) at a transcript level in the stellate ganglia sympathetic neurons. These data are shown against expression of other ion channel encoding genes, *SCN1A*-*SCN3A* (Na_v_) (Red), *KCNQ5* (K_v_) (Green) and CACNA1B (Ca_v_) (Blue). Here expression level indicates normalized count data. Here the count data per cell is divided by the total number of counts within that cell, multiplied by a scale factor and then natural-log transformed. **(B)** Immunohistochemistry highlights protein expression of Na_x_ in tyrosine hydroxylase positive stellate ganglia neurons. Cultured stellate ganglia cells were co-labelled for Na_x_ (Red), the sympathetic neuron marker, tyrosine hydroxylase (Green), and the nuclear stain Hoescht 33342 (Blue). **(C)** Knockdown of *SCN7A* significantly reduced the depolarization induced by an additional 30 mM NaCl, compared to *GAPDH* RNA knockdown (-2.87 ± 1.04 mV; *n* = 11–12; One-tailed unpaired *t*-test; *p* = 0.006) knockdown of a scrambled RNA control (–3.091 ± 0.8674 mV; *n* = 7–12; One-tailed unpaired *t*-test; *p* = 0.0012). **(D)** Knockdown of *SCN7A* caused a non-significant decrease in the increase in firing rate following 170 mM [NaCl]_o_ (4.5–0 Hz; n = 8–10; One-tailed Mann Whitney test; *p* = 0.06). Similarly, *SCN7A* knockdown reduced the firing rate induced by 170 mM [NaCl]_o_ when compared to a scrambled RNA knockdown control (4 Hz–0 Hz; n = 10; One-tailed Mann-Whitney test; *p* = 0.04).

With other mechanisms excluded, we then proceeded to assess the contribution of Na_x_, the molecular extracellular Na^+^ sensor. First, using a published single-cell RNA-sequencing dataset (Davis et al., 2020) we identified transcript expression of *SCN7A* (Na_x_) in a sympathetic stellate ganglia neuron neuron population (*TH*, *DBH* positive) (Figure 5A). For clarity, *SCN7A* expression is shown here in comparison with other key sympathetic ion channels that are known to be expressed in most sympathetic neurons, to show that the relative expression levels are similar. Protein expression was then confirmed via immunohistochemistry in stellate ganglia neurons (Figure 5B). Stellate ganglia neurons were co-labelled for Na_x_, tyrosine hydroxylase and the nuclear stain Hoescht 33342. The image was chosen to highlight the expression of Na_x_ in several sympathetic neurons simultaneously. Finally, we utilized siRNA mediated knockdown to reduce expression of *SCN7A* in stellate ganglia neurons *in vitro*. This approach significantly reduced 170 mM NaCl induced depolarization compared to *GAPDH* and scrambled RNA knockdown (Figure 5C) and prevented NaCl induced increases in firing rate when compared to GAPDH knockdown, but not scrambled RNA (Figure 5D). Notably the effect sizes are very similar in both instances (4.5 Hz and 4 Hz).

## Discussion

This study establishes a new role for post-ganglionic sympathetic neurons as sensors of extracellular Na^+^ concentration. To this end the study reports that: 1) Post-ganglionic sympathetic neurons from the superior cervical, stellate and superior mesenteric/coeliac ganglia, respond to elevated NaCl in a dose-dependent manner, 2) In stellate ganglia neurons we show that this response is independent of associated changes in extracellular Cl^−^ concentration and extracellular osmolarity, and is dependent upon extracellular Na^+^ concentration only, 3) Na_x_, an established Na^+^ sensor, is expressed in stellate neurons at a transcript and protein level, and appears to underly this response, whilst inhibition of other membrane Na^+^ pathways do not.

### Are Sympathetic Neurons Feasible Sensors?

The mechanism described here has a clear, dose dependent effect upon sympathetic ganglia neuron function, but the data presented do not contradict existing studies of central Na^+^ sensing. Instead, we suggest that an amplifying role for direct sympathetic neuron Na^+^ sensing. In this schema, activation of peripheral sympathetic ganglia neuron Na_x_ channels exaggerates preganglionic input, which in turn will be amplified due to activation of central sympathetic controlling regions such as the OVLT.

At this stage, we cannot rule out a dominant role for central Na^+^ sensors in the systemic response to elevated NaCl. For example, intracerebroventricular injection of NaCl recapitulates blood pressure elevations and increased SNA (Nomura et al., 2019), and central microinjections of inhibitors of paraventricular hypothalamic nucleus (PVN) activity prevented or altered systemic responses to NaCl (Badoer et al., 2003). However, we believe the evidence presented here suggests that peripheral sympathetic Na_x_ can also increase the gain of peripheral sympathetic stellate ganglia neurons, and therefore amplify central output. It is possible that in some cases direct activation of peripheral neurons may be enough to increase sympathetic responses. In support of this argument, inhibitors of PVN activity do not completely prevent heart rate elevations following intravenous (IV) NaCl (Badoer et al., 2003). Further work *in vivo* is required to resolve the relative contribution of central and peripheral Na^+^ sensing to exaggerated sympathetic responses, but as they share a common mechanism, Na_x_, both systems will likely operate within similar physiological constraints.

Most studies of Na_x_ utilize direct central delivery of NaCl, and in so doing exclude the activation of peripheral mechanisms. However, to our knowledge there exists three studies, which could be seen to contradict our findings. First, ablation of the entire Anteroventral third ventricular region (AV3V) region, including the OVLT, limits the impact of dietary NaCl on sciatic, vagal and aortic nerve induced reflexes (Simmonds et al., 2014). This well conducted study presents interesting findings, but it is worth noting that reflex activation may not reflect the increase in tonic SNA described by other authors. In the second case, high dietary NaCl caused a larger increase in splanchnic and renal SNA following glutamatergic stimulation of central sympathetic centers (Pawloski-Dahm and Gordon, 1993; Adams et al., 2007). The authors argued that this was consistent with increased central activity. However, these results could equally support increased gain at the level of the post-ganglionic sympathetic neuron, or a contribution of peripheral and central excitation. Finally, Antunes et al. (2006) demonstrated a reduction in osmolarity induced SNA following pre-collicular transection, which was abolished following removal of the circumventricular organs. In this study the authors used an *in-situ* preparation, where the lumbar nerves were exposed to standard Tyrode’s solution and not elevated NaCl. This preparation effectively prevents local sympathetic responses to high NaCl, and so it is unsurprising that the phenotype here is only central.

### Responses of Different Ganglia

NaCl sensitivity appears to be shared across post-ganglionic sympathetic neurons dissociated from the ganglia assessed here. This shared mechanism is of interest as functional variation has been reported between sympathetic ganglia (Jobling and Gibbins, 1999; Shanks et al., 2013). The sympathetic ganglia described here innervate many disparate target organs and tissues, and thus the effect of increasing sympathetic nerve activity would be expected to have equally wide-ranging effects. Prior *in vivo* studies suggest that alternate sympathetic ganglia may differentially respond to systemic, or central elevations in NaCl, although results are inconsistent.

Key targets of the stellate ganglia include the heart, skin and trachea and an expected hallmark of stellate ganglia activation is increased heart rate (Kummer et al., 1992; Rajendran et al., 2019). Our data suggest that stellate ganglia neurons, respond to elevated NaCl. However, prior studies *in vivo* measuring the effect of elevated NaCl on cardiac sympathetic function or cardiac parameters are variable, with studies suggesting either increased activity (Zhu et al., 2004; Kinsman et al., 2017a), no response (Gomes et al., 2017), or decreased heart rate (Pawloski-Dahm and Gordon, 1993) following elevated NaCl. The discrepancy between these results is likely due to the presence of the baroreflex *in vivo*, which reduces sympathetic nerve activity following an increase in blood pressure (Boscan et al., 2001). Inhibition of the baroreflex in animal models prevents decreases in heart rate following IV (Morita et al., 1991; Weiss et al., 1996) NaCl delivery and a similar mechanism has been described in humans (Piccirillo et al., 1996). In rodent models of hypertension, where the baroreflex is impaired (Ricksten and Thoren, 1980; Gordon and Mark, 1984), dietary NaCl causes a more consistent increase in heart rate (Simchon et al., 1989; Calhoun et al., 1994). Other studies also highlight an increase in cardiac depressor nerve responses (Pawloski-Dahm and Gordon, 1993; Simmonds et al., 2014) and increased baroreceptor gain (Trimarco et al., 1991), which could also alter cardiac responses *in vivo*. Under normal physiological conditions, reflexes regulating cardiac sympathetic function *in vivo* may override the excitatory effect observed here. However, upon the dampening or loss of these regulatory mechanisms, as observed in hypertension, elevated NaCl may increase cardiac sympathetic input via either central Na_x_ receptors or direct activation of stellate ganglia neurons as reported here. This theory explains the amplified heart rate in hypertensive models following increased dietary Na^+^ intake (Simchon et al., 1989; DiBona and Jones, 1991; Calhoun et al., 1994).

The superior mesenteric/coeliac ganglia innervate the kidneys, digestive system, pancreas and adrenal glands. Here, 170 mM NaCl induced an excitatory response in dissociated superior mesenteric/coeliac ganglia neurons, which contradicts the general decrease in renal SNA following experimental increases in serum NaCl *in vivo* observed by others (Badoer et al., 2003; Kinsman et al., 2017a; Kinsman et al., 2017b). Prior work (Morita et al., 1991) demonstrated that a combination of sinoaortic baroreceptor denervation, vagotomy and sectioning of the anterior and superior hepatic nerves eliminated a major component of the decrease in renal SNA following elevated NaCl. These data suggest that under normal physiological conditions *in vivo* the response of the renal nerves may be overridden by intrinsic reflexes to blood pressure increases via afferent sensory signaling and parasympathetic efferent feedback. A small component appears to be further inhibited through a mechanism involving PVN neurons (Badoer et al., 2003). Overall, suppressing renal SNA would favor natriuresis and act as a negative feedback to reduce NaCl (Morita et al., 1991; Sata et al., 2018). A physiological delay for this reflex may explain the small transient increase in renal SNA following elevated NaCl *in vivo* (Shi et al., 2007), with initial direct and/or central excitation of renal sympathetic nerves followed immediately by inhibition.

The superior cervical ganglia innervate structures including the iris, salivary glands, the carotid sinus, trachea several brainstem nuclei and large blood vessels of the cerebral vasculature (Flett and Bell, 1991; Kummer et al., 1992; Buller and Bolter, 1993). Activation of the SCG would cause for example iris dilation, sensitization of the baroreflex and vasoconstriction of cerebral arteries (Felder et al., 1983; Cassaglia et al., 2008; McDougal and Gamlin, 2015). Vasoconstriction of cerebral arteries has been reported in mechanically induced hypertension (Cassaglia et al., 2008), though to our knowledge neither iris dilation or changes in cerebral blood flow following high NaCl have been investigated.

As all of the tested ganglia all innervate a variety of vascular beds, activation of sympathetic ganglia in general may be responsible for the net vasoconstriction that follows elevated systemic NaCl (Howe et al., 1990).

### AVP as an Alternate Mechanism?

Given the tight control of plasma osmolarity through Arginine vasopressin (AVP), it is important to acknowledge its potential role in the maintenance of arterial blood pressure during NaCl intake. AVP is secreted from the midbrain via an osmolarity regulated mechanism, which is believed involve TRPV1 (Naeini et al., 2006). However, the relevance of AVP to NaCl induced elevations in blood pressure is controversial. Whilst Ribeiro et al. pharmacologically demonstrated a role for PVN AVP signaling in the response to elevated systemic Na^+^ concentration (Ribeiro et al., 2015), recent work highlighted that pharmacological inhibition of AVP receptors had no effect on increases in sympathetic activity following high NaCl, and only a minor effect on NaCl induced elevations in blood pressure (Nomura et al., 2019). Further, Nomura et al. report that Na_x_ knockout abolished most of the sympathetic and blood pressure response to elevated NaCl (Nomura et al., 2019), and it seems likely that the majority of the elevated SNS activity and subsequent increase in blood pressure occurs via a direct Na_x_ mediated mechanisms. Further, hypernatremic, Na_x_ mediated sympathetic and mean arterial blood pressure responses are greater than hyperosmotic, AVP mediated responses (Kinsman et al., 2017a; Kinsman et al., 2017b; Nomura et al., 2019).

Therefore, whilst, AVP, or perhaps other mechanisms such as angiotensin-II mediated sympathetic activation (Ma et al., 2001), may play a role *in vivo*, these observations support the primacy of Na_x_ mediated signaling such as the peripheral mechanism described here, and the previously described central mechanisms (Nomura et al., 2019).

### Limitations

We demonstrate that NaCl excites stellate ganglia neurons at concentrations as low as 150 mM, and other have also demonstrated a role of Na_x_ within the physiological range of Na concentrations. To interrogate the role of Nax, we deliberately chose a Na^+^ concentration of 170 mM, which is relatively high compared to the typical physiological range. This was chosen as it produced a large, robust response and helped avoid false negative results that may arise with more subtle changes. It is worth noting that whilst we have based the Na^+^ concentration range upon prior studies of Na_x_ (Hiyama et al., 2002) and the involvement of Na_x_ in hypertension (Nomura et al., 2019), there exists some controversy as to the extent of elevated Na^+^ concentration *in vivo* both in plasma and local interstitial fluid around neurons. However, global Na_x_ knockout has been reported to eliminate the blood pressure and sympathetic responses to elevated systemic Na^+^ concentration (Nomura et al., 2019), which must also operate under the biophysical constraints demonstrated here and previously (Hiyama et al., 2002). Therefore, the mechanism described here remains compatible with the current understanding of elevated Na^+^.

### Future Directions

Further study should continue this work *in vivo* although dissecting the peripheral role for Na_x_ will be challenging. A sympathetic neuron specific knockout of SCN7A may be informative. If the effect of elevated Na^+^ concentration upon sympathetic nerve activity and blood pressure *in vivo*, as demonstrated by Nomura et al. (2019), is diminished in the sympathetic specific *SCN7A* knockout model then this may resolve the individual contributions of sympathetic neuron Na_x_ and that in other neurons. However, it would not isolate the effect to the periphery or a specific sympathetic ganglion.

Current studies of Na_x_ function have also found that channel activation occurs at relatively high extracellular Na^+^ concentration. Given that global knockout of *SCN7A* appears to abolish the sympathetic nerve activity and blood pressure response to elevated serum Na^+^, it may be that the sensitivity of Na_x_ is shifted *in vivo* by one or more signaling mechanisms. Hiyama et al. (2013) previously demonstrated a role for endothelin-3 signaling in regulating central Na_x_ salt sensitivity in the context of thirst. Endothelin-3 signaling and other putative signaling mechanisms should be investigated in the central and peripheral nervous system in the context of hypertension.

Prior work has suggested that chronically elevated Na^+^ leads to decreases in the expression of tyrosine hydroxylase and noradrenaline transporter (Habecker et al., 2003). It would be of interest to resolve whether Na_x_ is involved in this process, or whether the expression of Na_x_ itself is affected by chronically raised extracellular Na^+^. Our preliminary data suggest that culturing sympathetic neurons for 24–36 h in 170 mM NaCl increases firing rate. This is likely to be activity dependent as it can blocked by culturing with TTX and 170 mM NaCl, although the transcriptional pathways responsible remain to be determined.

This study was performed in neurons cultured from male rats, which allowed for a more streamlined biophysical investigation. However, future work assessing the physiological role of this mechanism *in vivo* would benefit from an approach that used both male and female rats and investigated sex differences in peripheral Na_x_ responses.

## Summary and Conclusion

Using a variety of *in vitro* methodologies, we have established a molecular mechanism by which stellate ganglia neurons can sense raised extracellular Na^+^. The suggested mechanism, Na_x_, is in alignment with previous data demonstrating that systemic blood pressure and sympathetic nerve activity responses to raised serum Na^+^ are largely mediated by Na_x_. This study thereby opens further avenues of research on the role of peripheral Na^+^ sensing *in vivo*.

## Data Availability

The datasets presented in this study can be found in online repositories. The names of the repository/repositories and accession number(s) can be found below: https://www.ncbi.nlm.nih.gov/geo/, GSE144027.
